# Participant recruitment in sensitive surveys: a comparative trial of ‘opt in’ versus ‘opt out’ approaches

**DOI:** 10.1186/1471-2288-13-3

**Published:** 2013-01-11

**Authors:** Katherine J Hunt, Natalie Shlomo, Julia Addington-Hall

**Affiliations:** 1 Faculty of Health Sciences, University of Southampton, University Road, Southampton, SO17 1BJ, UK; 2 Cathie Marsh Centre for Census and Survey Research, University of Manchester, Humanities, Bridgeford Street, Manchester, M13 9PL, UK

**Keywords:** Recruitment, Survey research, Opt out, Sensitive research, Passive consent

## Abstract

**Background:**

Although in health services survey research we strive for a high response rate, this must be balanced against the need to recruit participants ethically and considerately, particularly in surveys with a sensitive nature. In survey research there are no established recommendations to guide recruitment approach and an ‘opt-in’ system that requires potential participants to request a copy of the questionnaire by returning a reply slip is frequently adopted. However, in observational research the risk to participants is lower than in clinical research and so some surveys have used an ‘opt-out’ system. The effect of this approach on response and distress is unknown. We sought to investigate this in a survey of end of life care completed by bereaved relatives.

**Methods:**

Out of a sample of 1422 bereaved relatives we assigned potential participants to one of two study groups: an ‘opt in’ group (n=711) where a letter of invitation was issued with a reply slip to request a copy of the questionnaire; or an ‘opt out’ group (n=711) where the survey questionnaire was provided alongside the invitation letter. We assessed response and distress between groups.

**Results:**

From a sample of 1422, 473 participants returned questionnaires. Response was higher in the ‘opt out’ group than in the ‘opt in’ group (40% compared to 26.4%: χ^2^ =29.79, p-value<.01), there were no differences in distress or complaints about the survey between groups, and assignment to the ‘opt out’ group was an independent predictor of response (OR=1.84, 95% CI: 1.45-2.34). Moreover, the ‘opt in’ group were more likely to decline to participate (χ^2^=28.60, p-value<.01) and there was a difference in the pattern of questionnaire responses between study groups.

**Conclusion:**

Given that the ‘opt out’ method of recruitment is associated with a higher response than the ‘opt in’ method, seems to have no impact on complaints or distress about the survey, and there are differences in the patterns of responses between groups, the ‘opt out’ method could be recommended as the most efficient way to recruit into surveys, even in those with a sensitive nature.

## Background

Survey research and self-completion questionnaires are an important way of gathering population-level information [1,2]. They rely on a high response rate to yield a representative sample without the loss of statistical power and introduction of response bias [3]. Response rate is therefore often regarded as the best single measure of the quality of survey data [4]. Survey response rates have been reducing over time [4-8] which increases the importance of using efficient recruitment strategies [9]. Therefore, recruitment procedures should aim to reduce response bias and increase response [3] at the same time as respecting participants’ rights [10].

Ethical recruitment of research participants is central to good research practice [11] and asking potential participants to provide written informed consent is considered to be the gold standard in recruitment [12]. Laws protecting the rights and privacy of the public have led to restrictions on the use of personal data and this has had an impact on research design and, in particular, recruitment [10,13,14]. For instance, the identification of potential research participants is more challenging if researchers are only permitted to approach individuals who formally ‘opt in’ to a study, thereby signalling an active willingness to participate [10]. Guidelines requiring potential participants to ‘opt in’ are based on recruitment practice in clinical research where the potential risk to participants is high. However, in observational research where the risk is lower, it has been argued that an ‘opt-out’ approach that uses passive consent is more efficient, whilst still maintaining choice to participate [12,15]. In our experience of conducting sensitive survey research with bereaved relatives, we have been required by ethics committees to use the ‘opt-in’ approach [16]. This involves sending an invitation to participate, and then only sending the questionnaire to those who positively respond to that invitation. This constitutes an extra step in survey recruitment that asks potential participants to give consent to be invited to participate in a survey, and then upon receipt of the questionnaire, to give passive consent by returning the completed copy.

Importantly, the impact of this approach on data quality is unknown even though it may challenge two important principles for survey researchers: the maintenance of scientific rigour by minimising bias; and the duty to uphold the principle of fully informed consent.

### Scientific rigour and bias

The ‘opt in’ method has been shown to be associated with reduced response. For instance, in a US multi-site survey, sites where the ‘opt in’ method was employed had a significantly lower response than areas where the ‘opt out’ method was used: 27% compared to 58% [17]. In our own bereavement surveys we have noted a considerable drop in response (from as high as 70% to as low as 35%) after switching to ‘opt in’ methods following research ethics committee decisions [16]. Our response rates are now lower than similar surveys that use traditional, ‘opt out’ methods [18]. Of course, surveys of the bereaved involve collection of sensitive data from people at a difficult time and this could impact negatively on response. However, there is evidence to suggest that people appreciate the opportunity to contribute to the delivery of end of life services by participating in research [11] and we have recorded a decline in response rates similar to the decline reported in other less sensitive surveys [4-8,16].

In an observational study that formally tested the effect of recruitment approach in a RCT, response was significantly higher in the ‘opt out’ group than the ‘opt in’ group [19]. Moreover, the ‘opt in’ group were significantly healthier and had less functional impairment than those who were recruited through the ‘opt out’ method. This may suggest that the extra step in recruitment creates a barrier to participation, threatening the validity of the findings. This consent bias, as it has been termed, can lead to over- or under-estimation of incidence/prevalence and may also mean that a study fails to detect true differences between groups (particularly between socio-economic and ethnic groups) [10] or fails to report the full range of experiences [14]. In satisfaction surveys (such as our survey of the quality of end of life care) it has been shown that non-responders are less likely to be satisfied than responders [20], thus posing a significant threat to the validity of findings [21].

### The principle of informed consent

It might be argued that in survey research the requirement for participants to ‘opt in’ without seeing the questionnaire obstructs the informed consent process. This approach does not provide potential participants with all the information they need to make an informed decision about participation. Although potential participants who ‘opt in’ are under no obligation to participate, they are not provided with enough information at the ‘opt in’ stage to make a sufficiently informed decision about whether to ‘opt in’ or not. In sensitive research such as our bereavement surveys, provision of the questionnaire with the initial invitation to participate is particularly important. We need to be open and forthcoming about the detailed nature participation by providing access to the questionnaire, not just the information sheet. When all the available information is present, a truly informed decision about participation can be made.

Although the ‘opt in’ method is favoured by most ethics committees based on experience with clinical trials, there is variability in decision-making for recruitment approach, particularly in the case of observational research [10,14,21]. However, some committees consider opting out to be acceptable [22] (something we have also noted in our own surveys) which is likely to result from a lack of clear guidelines for good recruitment practice in observational research [16]. Most importantly, neither recommendation seems to be based on solid evidence. The ethics of the ‘opt-in’ approach have not been formally investigated but preliminary research suggests that it contributes to lower response rates, a reduction in the validity of research findings and wasted time and resources [1,10,23].

Clearly, the impact of recruitment approach on distress and complaints is important if we are to uphold ethical practice and protect research participants. Indeed, approaching people during bereavement requires careful negotiation. Therefore, ahead of a national survey of the bereaved to capture views and experiences of end of life care, the English Department of Health commissioned a comparative trial of recruitment approach in two health districts. This would, for the first time, provide the evidence required to plan effective, efficient and ethically sensitive surveys and observational studies of end of life care.

We aimed, therefore, to investigate whether recruitment approach (‘opt in’ versus ‘opt out’) impacted upon response and response bias. We also sought to determine whether there was a difference in the number of people calling our bereavement telephone support line, or in the number of complaints between approaches.

## Methods

Data were collected as part of the IMPROVE survey, a survey of end of life care using VOICES, a questionnaire about the quality of end of life care services [16]. The English Office for National Statistics (ONS) extracted all deaths registered in two health districts in the south of England between October 2009 and April 2010. The included health districts were demographically distinct: one with an ethnically diverse and young population, the other with a predominantly white, older population.

Although we took a census of all deaths in the given time period (after excluding under 18s, deaths occurring ‘elsewhere’, and coroner-registered deaths) we used a self-weighting proportionally allocated stratified sample to assign potential participants to study group using the following strata: health district, age, sex, place of death (home, hospital, care home/hospice) and primary cause of death (cardiovascular disease, cancer, other causes). This ensured that both groups were equal with respect to the stratifying variables. All deaths were numbered consecutively and then the odd numbered records were assigned to the ‘opt out’ group and the even number records were assigned to the ‘opt in’ group. The study groups were as follows:

### Group 1 – the ‘opt out’ group

This group received a letter of invitation from ONS introducing the survey, a copy of the VOICES questionnaire (described below), a participant information sheet and a reply slip to request no further contact from ONS (respondents also had the opportunity to telephone ONS to make this request).

### Group 2 – the ‘opt in’ group

This group received a letter of invitation from ONS introducing the survey, the information sheet and a reply slip *to request the questionnaire* or to request no further contact or reminder letters from ONS (respondents also had the opportunity to telephone ONS to make this request).

Both groups had the opportunity to complete the questionnaire online using the same method. This meant that those in the ‘opt in’ group could avoid having to request the questionnaire by completing online. If potential participants did not decline or had not yet returned the questionnaire, two reminder letters were sent. The first was sent three weeks after the initial invitation, and the second reminder was sent five weeks after the initial invitation.

We set up a bereavement telephone support line so that participants could speak to a bereavement counsellor should they experience distress as a result of receiving the questionnaire. We recorded all calls to the bereavement support line as a measure of distress. We also kept a record of all complaints about the survey. Because of the sensitive nature of the questionnaire and because it is sent to bereaved relatives shortly after the death, previous VOICES surveys have sometimes received complaints about the nature of the survey. Participants wishing to make a complaint could do so by responding to the standard text in participant information sheets: ‘If you have a concern or a complaint about this study you should contact…’

#### Questionnaire items and supplementary data

The VOICES (Views of Informal Carers – Evaluation of Services) Short Form is a 58-item validated questionnaire completed by bereaved relatives [16]. Questionnaire items cover care provided by a range of health and social care practitioners across care settings. In addition to the questionnaire data, supplementary data on cause of death (ICD-10 codes), place of death, sex, age at death, ecological deprivation (Indices of Deprivation 2007), primary cause of death (ICD-10 codes), time since death, as well as sex and relation of informant (person who registered the death: potential participant) were obtained for the entire sample by the ONS.

Research ethics approval was sought from the University of Southampton, Faculty of Health Sciences Ethics Committee.

#### Statistical analyses

Using SPSS for Windows we assessed differences in characteristics between the two study groups: ‘opt in’ versus ‘opt out’. For nominal variables we used Chi Square tests to assess differences in proportions and for continuous variables we used t tests to assess the difference in means. Response for the total sample and for each study group was calculated and differences between the proportion responding between study groups was assessed with the Chi Square test. A significant chi-square test statistic tells us that the proportions across categories of a given variable are significantly different between the two study groups. To quantify the extra time taken for ‘opt in’ group respondents to request a copy of the questionnaire, we calculated time (in days) from the initial mail out to date of receipt of the completed questionnaire in both groups. We present time to response with median and interquartile range and used the Mann–Whitney U-Test to compare response time between study groups.

Logistic regression was used to determine predictors of response where ‘respond’ was the dependent variable and the supplementary data provided by the ONS formed the independent variables. The deviance of the model is presented based on the pseudo R^2^.

## Results

### Sample characteristics

2,272 deaths were registered in the two districts over the defined time period. Of these deaths, 788 were excluded because they were registered by a coroner (n=788), were classified as occurring ‘elsewhere’ (n=8), the decedent was aged under 18 years (n=13), or because the potential participant lived overseas (n=17). A further 24 deaths were subsequently excluded because the potential participant had changed address and the new address was unknown. The final sample included 1422 decedents and questionnaires were sent to the potential participant six to twelve months after the death of their relative.

Demographic and service use characteristics of the original sample are presented in Table 1. There was a higher proportion of female deaths in the sample; the majority were aged over 70 years; ‘other causes’ was the predominant cause of death; most died in a hospital; there were more female informants (potential participants) than male informants; and most informants were children of the deceased. There were no significant differences between study groups on any of the characteristics which was expected given the randomisation into the study groups.

**Table 1:** Characteristics of the sample and study groups

	**Whole sample frequency (%)**	**‘Opt in’ group frequency (%)**	**‘Opt out’ group frequency (%)**	**Test statistic and*p-value***
**Deceased sex**				χ^2^ =0.03, p-value=0.87
Male	617 (43.4)	310 (43.6)	307 (43.2)	
Female	805 (56.6)	401 (56.4)	404 (56.8)	
**Deceased age**				t=0.05, p-value= 0.96
Mean (SD)	80.3 (13.0)	80.3 (12.9)	80.3 (13.0)	
**Cause of death**				χ^2^ =0.08, p-value=0.96
CVD	354 (24.9)	176 (24.8)	178 (25.0)	
Cancer	485 (34.1)	245 (34.5)	240 (33.8)	
Other	583 (41.0)	290 (40.8)	293 (41.2)	
**Place of death**				χ^2^ =0.03, p-value=0.99
Home	195 (13.7)	98 (13.8)	97 (13.6)	
Hospital	725 (51.0)	361 (50.8)	364 (51.2)	
Care Home/Hospice	502 (35.3)	252 (35.4)	250 (35.2)	
**Deprivation quintile**				χ^2^ =2.79, p-value=0.59
1 (least deprived)	375 (26.4)	176 (24.8)	199 (28.0)	
2	294 (20.7)	156 (21.9)	138 (19.4)	
3	371 (26.1)	187 (26.3)	184 (25.9)	
4	331 (23.3)	168 (23.6)	163 (22.9)	
5 (most deprived)	51 (3.6)	24 (3.4)	27 (3.8)	
**Respondent sex**				χ^2^ =0.52, p-value=0.47
Male	643 (45.2)	315 (44.6)	328 (46.5)	
Female	768 (54.0)	391 (55.4)	377 (53.5)	
**Respondent relation to deceased**				χ^2^ =1.13, p-value=.57
Spouse/Partner	207 (14.6)	110 (16.7)	97 (14.7)	
Son/daughter	775 (54.5)	380 (57.7)	395 (60.0)	
Other	335 (23.6)	169 (25.6)	166 (25.2)	

### Response

61.3% of the sample (n=872/1422) responded to the invitation to participate in the survey in some way. 473 responders completed the questionnaire, either online or by returning a paper copy. Thus, the response across groups was 33%. Response was higher in the ‘opt in’ group (26.4% n=188/711) than in the ‘opt out’ group (40.0% n=285/711). This difference was significant (χ^2^ =29.79, p-value<.01).

Across the whole sample, 390 respondents formally declined to participate in the survey by returning the ‘reply slip’. The number of respondents returning this slip was higher in the ‘opt in’ group (240/711, 33.7%) than in the ‘opt out’ group (150/711, 21.1%) and this difference was significant (χ^2^=28.60, p-value<.01).

‘Overall response’, defined as all individuals who returned a reply slip to opt out of the survey or returned a completed questionnaire was 60.7% (n=863/1422). ‘Overall response’ was therefore similar in both groups (‘opt in’ group=428/711, 60.2%, ‘opt out’ group=435/711, 61.2%).

A small number of respondents in the ‘opt in’ group were lost to the recruitment process: 95.4% of those who requested a copy of the questionnaire returned a completed copy (188 out of 197 requests). The remaining 9 participants, although requesting a copy of the questionnaire, did not return a completed copy. The majority of those who formally declined to participate did not give a reason, but those who did suggested that the questionnaire was too distressing, the informant had registered the death in a professional capacity and did not know the decedent personally, the deceased had died suddenly or without receiving care.

There were no differences in the demographic characteristics of responders between the ‘opt in’ and ‘opt out’ groups (Table 2).

**Table 2:** Characteristics of responders in the ‘opt in’ and ‘opt out’ groups

	**‘Opt in’ group responders frequency (%)**	**‘Opt out’ group responders frequency (%)**	**Test statistic and p-value**
**Deceased sex**			χ^2^ =0.10, p-value=0.75
Male	72 (41.4)	106 (39.8)	
Female	102 (58.6)	160 (60.2)	
**Deceased age**			t=0.31, p-value=0.75
Mean (SD)	80.7 (11.7)	81.1 (12.8)	
**Cause of death**			χ^2^ = 0.99, p-value=0.61
CVD	45 (26.0)	61 (23.0)	
Cancer	69 (39.9)	102 (38.5)	
Other	59 (34.1)	102 (38.5)	
**Place of death**			χ^2^ =0.27, p-value=0.87
Home	29 (16.7)	47 (17.7)	
Hospital	74 (42.5)	117 (44.0)	
Care Home/Hospice	71 (40.8)	102 (38.3)	
**Deprivation quintile**			χ^2^ =3.57, p-value=0.47
1 (least deprived)	52 (30.1)	68 (25.7)	
2	40 (23.1)	57 (21.5)	
3	44 (25.4)	82 (30.9)	
4	34 (19.7)	48 (18.1)	
5 (most deprived)	3 (1.7)	10 (3.8)	
**Respondent sex**			χ^2^ =0.82, p-value=0.37
Male	52 (31.7)	89 (36.0)	
Female	112 (68.3)	158 (64.0)	
**Respondent relation to deceased**			χ^2^ =0.59, p-value=0.74
Spouse/Partner	42 (25.6)	64 (25.3)	
Son/daughter	93 (56.7)	151 (59.7)	
Other	29 (17.7)	38 (15.0)	

### Distress and complaints

We received no formal complaints about the survey, from either study group and there were only two calls to the bereavement telephone support line, one from each study group.

### Online completion

6.5% (n=93) of the sample completed the survey online, representing 19.6% of all responders. The online response was higher in the ‘opt in’ group (9.1% n=65, or 34.5% of the responders) compared to the ‘opt-out’ group (3.9% n=28, or 9.8% of the responders). This difference was significant (χ^2^ =15.80, p-value<.01).

### Response time

The median response time was higher in the ‘opt in’ group (24 days, IQR=18-59 days) than the ‘opt out’ group (16 days, IQR=7-24 days) and this difference was significant (U=8893.5, p-value<.01).

### Predictors of response

Assignment to the ‘opt out’ group was a significant independent predictor of response (OR=1.84) after adjustment for cause of death, place of death, months since death, age, informant sex, relation of informant and deprivation quintile (Table 3). After correction for these factors, it was more likely that a response was obtained for a hospital death (OR=1.40) and more likely if the responder was female (OR=1.71).

**Table 3:** Predictors of response using binary logistic regression

**Variable**	**Odds ratio**	**95% CI**
**Study group**	1.84	1.45-2.34
**Place of death**		
Home	0.87	0.60-1.26
Hospital	1.40	1.07-1.83
Care home/hospice	Reference category	Reference category
**Cause of death**		
CVD	0.92	0.68-1.24
Cancer	0.73	0.54-0.99
Other causes	Reference category	Reference category
**Months since death**	1.01	0.95-1.09
**Deprivation quintile**	1.06	0.96-1.17
**Age of decedent**	0.99	0.98-1.01
**Respondent sex**		
Male	Reference category	Reference category
Female	1.71	1.32-2.21
**Relation of respondent**		
Spouse/partner	1.27	0.75-1.71
Son/daughter	0.85	0.63-1.15
Other relative/friend	Reference category	Reference category

Pseudo-R^2^=.068.

Based on the model for the prediction of response in Table 3, we assessed the variation in the response propensities for the contrast between the respondents and non-respondents [24]. If there were no difference between the respondents and non-respondents, we would expect equal response propensities and no variation. In this case, we obtained 0.15 for the standard error of the response propensities where the maximum possible value is 0.5. Therefore, there appear to be differences between the characteristics of those responding and not responding to the study. We also decomposed this variation to obtain information on the characteristics that contributed the most to the contrast between respondents and non-respondents. Of the independent variables used in the model in Table 3, the Study Group contributes the most to the contrast between respondents and non-respondents followed by the respondent sex and place of death. The characteristics of individuals that are under-represented in the study are those of older ages in the ‘opt-out’ group who died from cancer in the home/care home, were least deprived and where the death was reported by a female family member.

### Differences in responses between study groups

Although there was no difference in the cause of death, place of death, deprivation status or ratings of care quality between the ‘opt in’ and ‘opt out’ responders, there were significant differences in responses for the questionnaire items pertaining to support for the participants themselves (as bereaved relatives) (Table 4). ‘Opt in’ participants were more likely to report receiving insufficient support to care for their relative at home (p-value<.05) and insufficient support at the actual time of death (p-value<.01). The differences in provision of bereavement support and staff sensitivity at the time of death approached significance.

**Table 4:** Questionnaire responses in study groups

**Questionnaire item**	**‘Opt in’ Group responders frequency (%)**	**‘Opt out’ group responders frequency (%)**	**χ**^**2**^**and*p-*value**
**Quality of out-of-hours care**			χ^2^ =1.67, p-value=0.62
Excellent	18 (23.7)	36 (27.5)	
Good	34 (44.7)	62 (47.3)	
Fair	15 (19.7)	17 (13.0)	
Poor	9 (11.8)	16 (12.2)	
**Quality of district nurse care**			χ^2^ =1.78, p-value=0.62
Excellent	31 (44.9)	52 (48.1)	
Good	30 (43.5)	41 (38.0)	
Fair	4 (5.8)	11 (10.2)	
Poor	4 (5.8)	4 (3.7)	
**Quality of GP care**			χ^2^ =1.86, p-value=.60
Excellent	31 (30.1)	63 (36.8)	
Good	43 (41.7)	65 (38.0)	
Fair	20 (19.4)	26 (15.2)	
Poor	9 (8.7)	17 (9.9)	
**Quality of hospital doctor care**			χ^2^ =1.68, p-value=0.64
Excellent	32 (30.5)	59 (38.1)	
Good	35 (33.3)	46 (29.7)	
Fair	27 (25.7)	34 (21.9)	
Poor	11 (10.5)	16 (10.3)	
**Quality of hospital nurse care**			χ^2^ =3.84, p-value=0.28
Excellent	31 (27.2)	63 (38.4)	
Good	39 (34.2)	49 (29.9)	
Fair	25 (21.9)	29 (17.7)	
Poor	19 (16.7)	23 (14.0)	
**Enough help and support to care for relative at home**			χ^2^=9.55, p-value=0.05
Yes	36 (45.6)	87 (64.9)	
Yes, but not as much as we wanted	26 (32.9)	25 (18.7)	
No, but we tried to get more	17 (21.5)	22 (16.4)	
**Dealt with in a sensitive manner after the death**			χ^2^ =2.45, p-value=0.09
Yes	137 (91.3)	223 (95.3)	
No	13 (8.7)	11 (4.7)	
**Enough help and support at the death**			χ^2^ =11.12, p-value=0.01
Yes, definitely	79 (52.7)	159 (67.7)	
Yes, to some extent	41 (27.1)	53 (22.6)	
No, not at all	30 (20.0)	23 (9.8)	
**Accessed bereavement support**			χ^2^ =4.81, p-value=0.09
Yes	18 (14.0)	34 (18.3)	
No	90 (69.8)	136 (73.1)	
No, but would have liked to	21 (16.3)	16 (8.6)	
**Respondent sex**			χ^2^ =0.52, p-value=0.47
Male	52 (31.7)	89 (36.0)	
Female	112 (68.3)	158 (64.0)	

## Discussion

To our knowledge, this is the first comparative trial of recruitment approach in surveys and as such, provides the first evidence that the ‘opt out’ approach is an efficient recruitment method for survey research. The ‘opt out’ approach is associated with a significantly higher response than the ‘opt in’ method, is a significant predictor of response, and is not associated with increased distress (calls to the bereavement support line) or complaints about the conduct or nature of the survey. Our data also support the argument that the ‘opt in’ approach is a more time-consuming method [1,25] by demonstrating a significantly shorter response time in the ‘opt out’ group.

Whilst only 80% of potential participants returned questionnaires in a Scottish survey where the ‘opt in’ method was imposed [1], we report that 95% of ‘opt in’ group responders returned questionnaires. In fact, we found that overall response, defined as correspondence with the survey administrators in whatever form, was the same in both study groups even though there was a significantly higher number of returned questionnaires in the ‘opt out’ group. The difference is explained by a much higher proportion of potential participants from the ‘opt in’ group declining to participate in the survey. This is an important finding that suggests that if potential participants are given a copy of the questionnaire with the initial invitation, they can make their decision to participate based on all the available information. This implies that it is fully informed decision-making that determines participation in survey research, which is congruent with established drivers of survey participation [26]. A general assumption made by the ‘opt in’ approach is that non-responders have withheld consent [1]. However, research suggests that non-response is more likely to result from apathy, lack of interest [3] or misconceptions about the aims of the study [27]. Therefore, one might suggest that the ‘opt in’ method could lead to misconceptions or an incomplete understanding of a study’s aims. Indeed, the ‘opt in’ approach asks potential participants to express an interest in a study that they are insufficiently informed about. Again, our finding that overall response was the same in both groups, yet questionnaire completion was higher when a copy of the questionnaire was sent with the initial invitation, supports this view.

Previous research has suggested that recruitment approach is associated with responses to individual questionnaire items [14,25] and our findings would support this. Although our survey is limited by its reliance on proxy reports which means that respondents are not commenting on their own care (which may explain why we found no differences in the rated quality of care between study groups), significant between-group differences were observed for the variables that relate to care that the respondent received: ‘opt in’ group responders were more likely to be dissatisfied with support in helping them to care for their relative at home and support provided at the time of the death. This suggests that care experiences may play a role in response and the ‘opt in’ method might discourage response among those who were satisfied or indifferent about the care they received. The fact that there were no differences in the demographic characteristics of responders in each study group supports this finding. However, these findings are not consistent with previous research which has suggested that responders to satisfaction surveys are more likely to be satisfied than non-responders [20]. Of course, these findings may be explained by the population studied: people experience considerable distress when a relative is dying and if it is felt that end of life care services fail to provide the care and support that is needed at that time, anger may be expressed [28]. It is therefore possible that, unlike in other healthcare satisfaction surveys, it is dissatisfaction, rather than satisfaction, that drives response in bereavement surveys.

Our trial builds on work by Junghans *et al*. [19] where telephone calls were used to recruit the ‘opt out’ group whilst a letter of invitation was issued to the ‘opt in’ group. Although this is consistent with data restrictions, it meant that different approaches were used for each group, and because we know that personal contact increases response [26], it is likely that the differences in response were exaggerated as a result of this disparity. We contacted both groups using the same medium and noted a more marked difference in the response between groups than in the trial by Junghans *et al*.

Our trial has a number of important limitations. Firstly, because it was nested within a survey of bereaved relatives, the findings may not be generalizable to other survey populations. Bereaved relatives form a distinct group and their motivations for survey participation may be very different to respondents to other surveys. In addition, the survey was completed by proxies: it was not the end of life care recipient who completed the questionnaire, but a relative on their behalf. Patterns in response, and predictors of response, may be different if people are reporting on their own care. Indeed, we noticed a difference in response patterns to questionnaire items when respondents were considering care they received themselves, rather than care received by their deceased relative. The number of formal complaints about the research and calls to the bereavement support telephone line are only proxy measures of distress and so our conclusion that the ‘opt out’ approach is not associated with greater levels of distress may not necessarily be based upon all respondents’ experiences. It is possible that distressed respondents simply did not contact either service. Finally, we can only make recommendations for optimising recruitment strategies for survey research, and not observational studies in general. Further research is required to inform recruitment practice in other study designs.

## Conclusion

We report that the imposition of recruitment recommendations designed for clinical trials on observational research can adversely affect the scientific integrity of health surveys. Our findings approve the ‘opt out’ method of recruitment by demonstrating that it leads to higher response, provides participants with all the information they require to make an informed decision about participation, and does not seem to be associated with higher levels of distress than the ‘opt in’ method, even in sensitive surveys.

## Competing interest

All authors declare that they have no competing interest.

## Authors’ contributions

KH carried out data collection and analysed the data with NS. JAH designed the survey. All authors read and approved the final manuscript.

## Pre-publication history

The pre-publication history for this paper can be accessed here:

http://www.biomedcentral.com/1471-2288/13/3/prepub

## References

[B1] Angus VC Entwistle VA Emslie MJ Walker KA Andrew JE The requirement for prior consent to participate on survey response rates: a population-based survey in Grampian BMC Health Serv Res 2003 3 21 10.1186/1472-6963-3-21 14622444 PMC293468

[B2] Cartwright A Health surveys in practice and in potential 1983 London: King’s Fund Publishing Office

[B3] Iverson A Liddell K Fear N Hotopf M Wessely S Consent, confidentiality, and the Data Protection Act Br Med J 2006 332 165 169 10.1136/bmj.332.7534.165 16424496 PMC1336771

[B4] Biener L Garrett CA Gilpin EA Roman AM Currivan DB Consequences of declining survey response rates for smoking prevalence estimates Am J Prev Med 2004 27 254 257 10.1016/j.amepre.2004.05.006 15450639

[B5] Steeh CG Trends in nonresponse rates, 1952–1979 Pub Opinion Quart 1981 45 40 57 10.1086/268633

[B6] Kiezebrink K Crombie I Irvine L Swanson V Power K Wrieden W Slane P Strategies for achieving a high response rate in a home interview survey BMC Med Res Methodology 2009 9 46 10.1186/1471-2288-9-46 PMC2709923 19566931

[B7] Baruch Y Holtom B Survey response rate levels and trends in organizational research Human Relations 2008 61 1139 1160 10.1177/0018726708094863

[B8] Galea S Tracy M Participation rates in epidemiologic studies Ann Epidemiol 2007 17 643 653 10.1016/j.annepidem.2007.03.013 17553702

[B9] Kelley K Clark B Brown V Sitzia J Good practice in the conduct and reporting of survey research Int J Qual Health Care 2003 15 261 266 10.1093/intqhc/mzg031 12803354

[B10] Hewison J Haines A Overcoming barriers to recruitment in health research Br Med J 2006 333 300 302 10.1136/bmj.333.7562.300 16888308 PMC1526952

[B11] Duke S Bennett H A narrative review of the published ethical debates in palliative care research and an assessment of the adequacy to inform research governance Pall Med 2010 24 111 126 10.1177/0269216309352714 19965950

[B12] Vellinga V Cormican M Hanahoe B Bennett K Murphy AW Opt-out as an acceptable method of obtaining consent in medical research: a short report BMC Med Res Methodology 2011 11 40 10.1186/1471-2288-11-40 PMC3079702 21470399

[B13] Gardiner C Barnes S Small N Gott M Payne S Seamark D Halpin D Reconciling informed consent and ‘do no harm’: ethical challenges in palliative care research and practice in chronic obstructive pulmonary disease Pall Med 2010 24 468 472 10.1177/0269216310367536 20444768

[B14] Jungans C Jones M Consent bias in research: how to avoid it Heart 2007 93 1024 1025 10.1136/hrt.2007.120113 17699171 PMC1955004

[B15] Clark AM Jamieson R Findlay IN Registries and informed consent N Engl J Med 2004 351 612 614 15295059 10.1056/NEJM200408053510621

[B16] Hunt KJ Shlomo N Richardson A Addington-Hall JM VOICES redesign and testing to inform a national end of life care survey 2011 Southampton: University of Southampton

[B17] Nelson K Garcia RE Brown J Mangione CM Louis TA Keelier E Cretin S Do patient consent procedures affect participation rates in health services research? Med Care 2002 40 283 288 10.1097/00005650-200204000-00004 12021684

[B18] Ingleton C Morgan J Hughes P Noble B Evans A Clark D Carer satisfaction with end of life care in Powys, Wales: a cross-sectional survey Health Soc Care Community 2004 12 43 52 10.1111/j.1365-2524.2004.00467.x 14675364

[B19] Junghans C Feder G Hemingway H Timmis A Jones M Recruiting patients to medical research: double blind randomised trial of ‘opt in’ versus ‘opt out’ strategies Br Med J 2005 331 940 10.1136/bmj.38583.625613.AE 16157604 PMC1261191

[B20] French K Methodological considerations in hospital patient opinion surveys Int J Nurs Stud 1981 18 7 32 10.1016/0020-7489(81)90004-3 6906348

[B21] Koh ME Duffett M Willison DJ Written informed consent and selection bias in observational studies using medical records: systematic review Br Med J 2009 338 b866 10.1136/bmj.b866 19282440 PMC2769263

[B22] Pegg MS Overcoming barriers to health research: some research ethics committees believe in facilitating health research Br Med J 2006 333 398 16916840 10.1136/bmj.333.7564.398-a PMC1550442

[B23] Walley T Using personal health information in medical research Br Med J 2006 332 130 132 10.1136/bmj.332.7534.130 16424468 PMC1336750

[B24] Shlomo N Schouten B Skinner CJ Estimation of an indicator of the representativeness of survey response Journal of Statistical Planning and Inference 142 2012 201 211

[B25] Boardman HF Thomas E Ogden H Croft PR Millson DS A method to determine if consenters to population surveys are representative of the target population J Public Health Med 2005 27 129 137 10.1093/pubmed/fdi017 15774566

[B26] Edwards P Roberts I Clarke M diGiuseppe C Pratap S Wentz R Kwan I Increasing response to postal questionnaires: systematic review Br Med J 2002 324 1183 10.1136/bmj.324.7347.1183 12016181 PMC111107

[B27] Crombie IK McMurdo MET Overcoming barriers to recruitment in health research: concerns of individual participants need to be dealt with Br Med J 2006 333 398 16916841 10.1136/bmj.333.7564.398 PMC1550468

[B28] Ellershaw J Ward C Care of the dying patient: the last hours or days of life Br Med J 2003 326 7379 30 34 10.1136/bmj.326.7379.30 12511460 PMC1124925

